# Supplementary material for: Adult dengue vaccination in a low transmission setting: A modelling study in Singapore
Source: PLoS Negl Trop Dis. 2026 Mar 2;20(3):e0014062. doi: 10.1371/journal.pntd.0014062 (PMC12970969; doi:10.1371/journal.pntd.0014062)
Supplement: S1 Text — (DOCX) [file pntd.0014062.s001.docx]

**S1 Text**

**Adult dengue vaccination in a low transmission setting: A modelling study in Singapore**

Abhishek Senapati^1*^, Tun-Linn Thein^2^, Yee-Sin Leo^1,2,3,4,5^, Hannah Eleanor Clapham^1*^

^1^Saw Swee Hock School of Public Health, National University of Singapore and National University Health System, Singapore

^2^National Centre for Infectious Diseases, Singapore

^3^Department of Infectious Diseases, Tan Tock Seng Hospital, Singapore

^4^Lee Kong Chian School of Medicine, Nanyang Technological University, Singapore

^5^Department of Medicine, Yong Loo Lin School of Medicine, National University of Singapore, Singapore

^*^Corresponding authors: [abhisena@nus.edu.sg](mailto:abhisena@nus.edu.sg); <hannah.clapham@nus.edu.sg>

**Contents**

[Mathematical equations of the transmission model 2](#_Toc219279613)

[Estimation of annual force of infection 15](#_Toc219279614)

[Dengue surveillance data 17](#_Toc219279615)

[Model fitting 18](#_Toc219279616)

[Hospitalization rate 20](#_Toc219279617)

[Serotype distribution of dengue infection under dominating serotype scenarios 21](#_Toc219279618)

[Sensitivity analysis 22](#_Toc219279619)

[Number of dengue cases and hospitalizations averted per 1000 vaccination 24](#_Toc219279620)

[Impact of vaccination in different age groups 25](#_Toc219279621)

[Impact of vaccination with alternative targeted age-groups 27](#_Toc219279622)

[References 29](#_Toc219279623)

# **Mathematical equations of the transmission model**

$$\begin{aligned} \frac{dS^{a}}{dt}=\Lambda^{h}-\left( \sum_{i=1}^{4} \lambda_{i}^{m}+\mu^{h} \right)S^{a}+a_{+}S^{a-1}-a_{-}S^{a}-v_{S^{a}},\# \end{aligned}$$

$$\begin{aligned} \frac{dI_{i}^{a}}{dt}=\lambda_{i}^{m}S^{a}-\left( \gamma+\mu^{h} \right)I_{i}^{a}+a_{+}I_{i}^{a-1}-a_{-}I_{i}^{a},\# \end{aligned}$$

$$\begin{aligned} \frac{dC_{i}^{a}}{dt}=\gamma I_{i}^{a}-\left( \alpha+\mu^{h} \right)C_{i}^{a}+a_{+}C_{i}^{a}-a_{-}C_{i}^{a}-v_{C_{i}^{a}},\# \end{aligned}$$

$$\begin{aligned} \frac{dS_{i}^{a}}{dt}=\alpha C_{i}^{a}-\left( \sum_{j\neq i} \lambda_{j}^{m}+\mu^{h} \right)S_{i}^{a}+a_{+}S_{i}^{a-1}-a_{-}S_{i}^{a}-v_{S_{i}^{a}},\# \end{aligned}$$

$$\begin{aligned} \frac{dI_{ij}^{a}}{dt}=\lambda_{j}^{m}S_{i}^{a}-\left( \gamma+\mu^{h} \right)I_{ij}^{a}+a_{+}I_{ij}^{a-1}-a_{-}I_{ij}^{a},\# \end{aligned}$$

$$\begin{aligned} \frac{dR^{a}}{dt}=\gamma\sum_{i,j} I_{ij}^{a}-\mu^{h}R^{a}+a_{+}R^{a-1}-a_{-}R^{a}-v_{R^{a}},\#\left( 1 \right) \end{aligned}$$

$$\begin{aligned} \frac{dS^{m}}{dt}=\Lambda^{m}-\left( \sum_{i=1}^{4} \lambda_{i}^{h}+\mu^{m} \right)S^{m},\# \end{aligned}$$

$$\begin{aligned} \frac{dE_{i}^{m}}{dt}=\lambda_{i}^{h}S^{m}-\left( \sigma^{m}+\mu^{m} \right)E_{i}^{m},\# \end{aligned}$$

$$\begin{aligned} \frac{dI_{i}^{m}}{dt}=\sigma^{m}E_{i}^{m}-\mu^{m}I_{i}^{m},\# \end{aligned}$$

$$\begin{aligned} \frac{dS_{v}^{a}}{dt}=v_{S^{a}}-\left( 1-\epsilon^{inf-} \right)\left( \sum_{i=1}^{4} \lambda_{i}^{m}+\mu^{h} \right)S_{v}^{a}+a_{+}S_{v}^{a-1}-a_{-}S_{v}^{a},\# \end{aligned}$$

$$\begin{aligned} \frac{dI_{vi}^{a}}{dt}=\left( 1-\epsilon^{inf-} \right)\lambda_{i}^{m}S_{v}^{a}-\left( \gamma+\mu^{h} \right)I_{vi}^{a}+a_{+}I_{vi}^{a-1}-a_{-}I_{vi}^{a},\# \end{aligned}$$

$$\begin{aligned} \frac{dC_{vi}^{a}}{dt}=v_{C_{i}^{a}}+\gamma I_{vi}^{a}-\left( \alpha+\mu^{h} \right)C_{vi}^{a}+a_{+}C_{vi}^{a-1}-a_{-}C_{vi}^{a},\# \end{aligned}$$

$$\begin{aligned} \frac{dS_{vi}^{a}}{dt}=v_{S_{i}^{a}}+\alpha C_{vi}^{a}-\left( 1-\epsilon_{s}^{inf+} \right)\left( \sum_{j\neq i} \lambda_{j}^{m}+\mu^{h} \right)S_{vi}^{a}+a_{+}S_{vi}^{a-1}-a_{-}S_{vi}^{a},\# \end{aligned}$$

$$\begin{aligned} \frac{dI_{vij}^{a}}{dt}=\left( 1-\epsilon^{inf+} \right)\lambda_{j}^{m}S_{vi}^{a}-\left( \gamma+\mu^{h} \right)I_{vij}^{a}+a_{+}I_{vij}^{a-1}-a_{-}I_{vij}^{a},\# \end{aligned}$$

$$\begin{aligned} \frac{R_{v}^{a}}{dt}=v_{R^{a}}+\gamma\sum_{i,j} I_{vij}^{a}-\mu^{h}R_{v}^{a}+a_{+}R_{v}^{a-1}-a_{-}R_{v}^{a}.\# \end{aligned}$$

The force of infection on humans due to serotype *i,* is given by

$$\begin{aligned} \lambda_{i}^{m}=\frac{b\beta_{i}^{m}I_{i}^{m}}{N}.\#\left( 2 \right) \end{aligned}$$

The force of infection on mosquitoes from human infected with serotype *i*, is given by

$$\begin{aligned} \lambda_{i}^{h}=\frac{b\beta_{i}^{h}\left( \sum_{a} I_{i}^{a}+\sum_{a,j\left( j\neq i \right)} I_{ji}^{a}+\sum_{a} I_{vi}^{a}+\sum_{a,j\left( j\neq i \right)} I_{vji}^{a} \right)}{N}.\#\left( 3 \right) \end{aligned}$$

The incidence of infection of age *a* at time *t* due to serotype *i* is given by,

$$\begin{aligned} Inf_{i}^{a}\left( t \right)=\lambda_{i}^{m}S^{a}+\left( 1-\epsilon^{inf-} \right)\lambda_{i}^{m}S_{v}^{a}+\sum_{j\left( \neq i \right)} \lambda_{i}^{m}S_{j}^{a}+\sum_{j\left( \neq i \right)} \left( 1-\epsilon^{inf+} \right)\lambda_{i}^{m}S_{vj}^{a}.\#\left( 4 \right) \end{aligned}$$

The incidence of reported cases of age *a* at time *t* due to serotype *i* is given by,

$$\begin{aligned} Rep_{i}^{a}\left( t \right)=\rho_{1}^{a}\lambda_{i}^{m}S^{a}+\rho_{2}^{a}\sum_{j\left( \neq i \right)} \lambda_{i}^{m}S_{j}^{a}+\rho_{1}^{a}\left( 1-\epsilon_{i}^{vcd-|inf-} \right)\left( 1-\epsilon^{inf-} \right)\lambda_{i}^{m}S_{v}^{a}+ \\ \rho_{2}^{a}\sum_{j\left( \neq i \right)} \left( 1-\epsilon_{i}^{vcd+|inf+} \right)\left( 1-\epsilon^{inf+} \right)\lambda_{i}^{m}S_{vj}^{a}.\#\left( 5 \right) \end{aligned}$$

The incidence of hospitalization of age *a* at time *t* due to serotype *i* is given by,

$$\begin{aligned} Hosp_{i}^{a}\left( t \right)=\xi_{1}\rho_{1}^{a}\lambda_{i}^{m}S^{a}+\xi_{2}\rho_{2}^{a}\sum_{j\left( \neq i \right)} \lambda_{i}^{m}S_{j}^{a}+ \\ \xi_{1}\rho_{1}^{a}\left( 1-\epsilon_{i}^{hosp-|vcd-} \right)\left( 1-\epsilon_{i}^{vcd-|inf-} \right)\left( 1-\epsilon^{inf-} \right)\lambda_{i}^{m}S_{v}^{a}+ \\ \xi_{2}\rho_{2}^{a}\sum_{j\left( \neq i \right)} \left( 1-\epsilon_{i}^{hosp+|vcd+} \right)\left( 1-\epsilon_{i}^{vcd+|inf+} \right)\left( 1-\epsilon^{inf+} \right)\lambda_{i}^{m}S_{vj}^{a}.\#\left( 6 \right) \end{aligned}$$

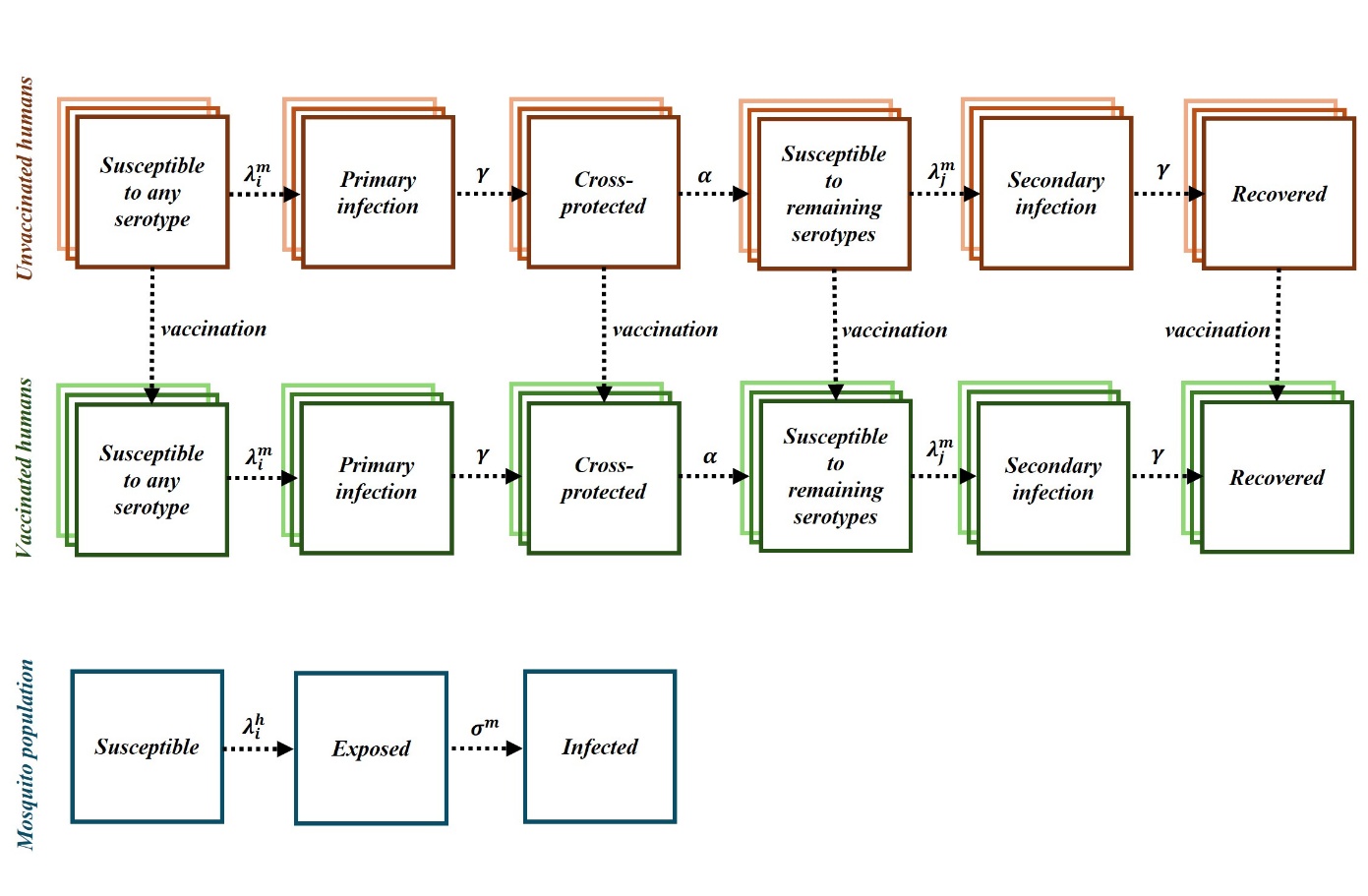


**Fig A**: Simplified schematic of the transmission model. Human compartments are stratified by age group, and demographic transitions are omitted for clarity. The force of infection on humans due to serotype i ($\lambda_{i}^{m}$), and the force of infection on mosquitoes due to serotype i ($\lambda_{i}^{h}$) are described in equation 2 and equation 3 respectively. Descriptions of all variables and parameters are provided in Table A and Table B respectively.

| **Symbol** | **Description** |
| --- | --- |
| $S^{a}\left( t \right)$ | Number of people of age *a* at time *t*, who are susceptible to infection from any serotype |
| $I_{i}^{a}\left( t \right)$ | Number of people of age *a* at time *t*, who are infected with serotype *i* (primary infection) |
| $C_{i}^{a}\left( t \right)$ | Number of age *a* at time *t* who are immune to infection from serotype *i*, and temporarily protected against heterologous infection |
| $S_{i}^{a}\left( t \right)$ | Number of people of age a at time *t* who are immune to infection from serotype *i* but remain susceptible to infection from other serotypes |
| $I_{ij}^{a}\left( t \right)$ | Number of people of age a at time *t* who are immune to infection from serotype *i,* but infected with serotype *j* ($\neq i)$ (secondary infection) |
| $R^{a}\left( t \right)$ | Number of people of age a at time t who are recovered from secondary infection |
| $S_{v}^{a}\left( t \right)$ | Number of vaccinated people of age *a,* at time *t*, who are susceptible to infection from any serotype |
| $I_{vi}^{a}\left( t \right)$ | Number of vaccinated people of age *a,* at time *t*, who are infected with serotype *i* (primary infection) |
| $C_{vi}^{a}\left( t \right)$ | Number of vaccinated people of age *a* at time *t* who are immune to infection from serotype *i*, and temporarily protected against heterologous infection |
| $S_{vi}^{a}\left( t \right)$ | Number of vaccinated people of age *a* at time *t* who are immune to infection from serotype *i* but remain susceptible to infection from other serotypes |
| $I_{vij}^{a}\left( t \right)$ | Number of vaccinated people of age *a* at time *t* who are immune to infection from serotype *i,* but infected with serotype *j* ($\neq i)$ (secondary infection) |
| $R_{v}^{a}\left( t \right)$ | Number of vaccinated people of age *a* at time *t* who are recovered from secondary infection |
| $N\left( t \right)$ | Total human population at time *t* |
| $S^{m}\left( t \right)$ | Number of uninfected adult female mosquitoes at time *t* |
| $E_{i}^{m}\left( t \right)$ | Number of adult female mosquitoes at time *t* in incubation period infected with serotype *i* |
| $I_{i}^{m}\left( t \right)$ | Number of infectious adult female mosquitoes infected with serotype *i* |

**Table A:** The description of the state variables used in the transmission model.

| **Parameters** | **Description** | **Value** | **Source** |
| --- | --- | --- | --- |
| $\Lambda^{h}$ | Human recruitment rate | Birth rate×Total population  (Time dependent) | [1,2] |
| $\mu^{h}$ | Mortality rate of human | Time dependent | [1] |
| $\rho_{2}^{a}$ | Fraction of secondary infection in age *a* to be reported | Table D | Estimated from age-stratified annual incidence of reported dengue cases per 100,000 population from 2014-2020 |
| $\rho_{1}^{a}$ | Fraction of primary infection in age *a* to be reported | $\frac{1}{2}\rho_{2}^{a}$ | [3,4] |
| $\xi_{2}^{a}$ | Fraction of reported cases from secondary infection in age $a$ requires hospitalization | Table E | [5] |
| $\xi_{1}^{a}$ | Fraction of reported cases from primary infection in age $a$ requires hospitalization | $\frac{1}{4}\xi_{2}$ | [3,4] |
| $\frac{1}{\gamma}$ | Infectious period of human | 4 days | [6] |
| $\frac{1}{\alpha}$ | Duration of heterologous protection from infection | 1 year | [7] |
| $b$ | Mosquito biting rate | 15 | Assumed |
| $\beta_{i}^{h}$ | Per bite probability of transmission from infected human to mosquitoes | 0.2 | Assumed |
| $\beta_{i}^{m}$ | Per bite probability of transmission from infected mosquitoes with *i*th serotype  to human | 0.166; 0.176; 0.152; 0.144 | Calibrated to annual dengue FOI estimates |
| $\frac{1}{\sigma^{m}}$ | Mean extrinsic incubation period | 10 days | [8,9] |
| $\Lambda^{m}$ | Recruitment rate of mosquito | $\mu^{m}$×Total mosquito pop. |  |
| $\mu^{m}$ | Adult mosquito mortality rate | 0.1 per day | [3,10] |
| $a_{+}$ | Rate at which individuals enter age group *a* from age group *a-1* | $\frac{1}{365}$ per day for *a = 2, 3,…,91,*  and 0 for *a =1* |  |
| $a_{-}$ | Rate at which individuals leave age group *a* and enters age group *a+1* | $\frac{1}{365}$ per day for *a = 1,3,…,90,*  and 0 for *a =91* |  |
| $v_{x}$ | Vaccination rate in compartments $x(=S^{a}, C_{i}^{a}, S_{i}^{a}, R^{a})$ | These rates have been adjusted in such a way that coverage (given) in a targeted age group is maintained. We assumed the coverage to be 20%, 50% and 80% |  |
| $\epsilon^{inf+}$ | Efficacy of vaccine against infection due to any serotype, among baseline seropositive individuals | 9·3% (95% CI: –35·9–38·8) | [11]. |
| $\epsilon^{inf-}$ | Efficacy of vaccine against infection due to any serotype, among baseline seronegative individuals | 48·1% (95% CI: 35·2–58·5) | [11] |
| $\epsilon_{i}^{vcd+}$ | Efficacy of vaccine against VCD due to serotype *i*, among baseline seropositive individuals | Table C |  |
| $\epsilon_{i}^{vcd-}$ | Efficacy of vaccine against VCD due to serotype *i*, among baseline seronegative individuals | Table C |  |
| $\epsilon_{i}^{hosp+}$ | Efficacy of vaccine against hospitalization due to serotype *i*, among baseline seropositive individuals | Table C |  |
| $\epsilon_{i}^{hosp-}$ | Efficacy of vaccine against hospitalization due to serotype *i*, among baseline seronegative individuals | Table C |  |
| $\epsilon_{i}^{vcd+\mid inf+}$ | Efficacy of vaccine against VCD given infection due to serotype i, among baseline seropositive individuals | $\frac{\epsilon_{i}^{vcd+}-\epsilon^{inf+}}{1-\epsilon^{inf+}}$ |  |
| $\epsilon_{i}^{vcd-\mid inf-}$ | Efficacy of vaccine against VCD given infection due to serotype *i*, among baseline seronegative individuals | $\frac{\epsilon_{i}^{vcd-}-\epsilon^{inf-}}{1-\epsilon^{inf-}}$ |  |
| $\epsilon_{i}^{hosp+\mid vcd+}$ | Efficacy of vaccine against hospitalization given VCD due to serotype *i*, among baseline seropositive individuals | $\frac{\epsilon_{i}^{hosp+}-\epsilon_{i}^{vcd+}}{1-\epsilon_{i}^{vcd+}}$ |  |
| $\epsilon_{i}^{hosp-\mid vcd-}$ | Efficacy of vaccine against hospitalization given VCD due to serotype *i*, among baseline seronegative individuals | $\frac{\epsilon_{i}^{hosp-}-\epsilon_{i}^{vcd-}}{1-\epsilon_{i}^{vcd-}}$ |  |

**Table B:** Description of the model parameters used in the transmission model.

| **Vaccine efficacy against** | **Serotype** | **Seropositive** | **Seronegative** |
| --- | --- | --- | --- |
| VCD | DENV-1 | 56·1  (44·6–65·2) | 45·4  (26·1–59·7) |
|  | DENV-2 | 80·4  (73·1–85·7) | 88·1  (78·6–93·3) |
|  | DENV-3 | 52·3  (36·7–64·0) | –15·5  (–108·2–35·9) |
|  | DENV-4 | 70·6  (39·9–85·6) | –105·6  (–628·7–42·0) |
| Hospitalization | DENV-1 | 66·8  (37·4–82·3 | 78·4  (43·9–91·7) |
|  | DENV-2 | 95·8  (89·6–98·3) | **88·1**  **(78·6–93·3)** |
|  | DENV-3 | 74·0  (38·6–89·0) | –87·9  (–573·4–47·6) |
|  | DENV-4 | **70·6**  **(39·9–85·6)** | **–105·6**  **(–628·7–42·0)** |

**Table C:** Vaccine efficacy estimates of Qdenga in the safety set, approximately 57 months after the first dose, against virologically confirmed dengue (VCD) and hospitalization, stratified by baseline serostatus, serotype**.** The efficacy estimates against hospitalizations due to DENV-2 among seronegative individuals, due to DENV-4 among both seropositive and seronegative individuals are assumed to be same (highlighted in bold) as the corresponding efficacy estimates against VCD, as they were not estimable during the trials.


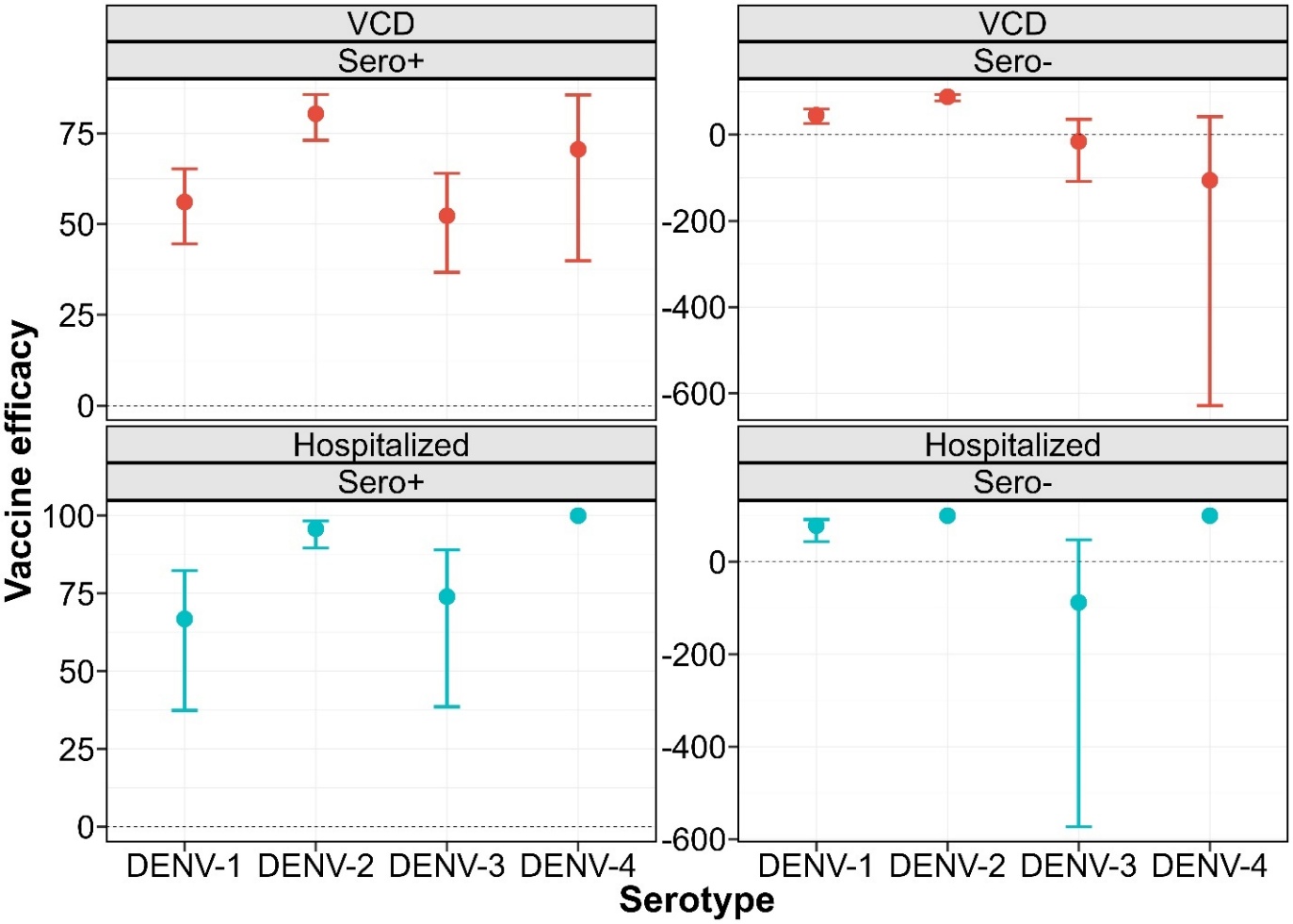


**Fig B:** The serotype (DENV-1-4) and baseline serostatus (seropositive (Sero+), seronegative (Sero-)) stratified vaccine efficacy estimates of Qdenga against VCD and hospitalization approximately 57 months after the first dose of the vaccine. The dots represent the point estimates, and the error bar represents the 95% confidence interval. The horizontal dashed line denotes the zero-vaccine efficacy. The efficacy estimates against hospitalization among seropositive due to DENV-4, among seronegative due to DENV-2 and DENV-4, were not estimable due to zero incidence among individuals in vaccine group. All the data have been reported in [12].

# **Estimation of annual force of infection**

The fraction of people who remain susceptible (0-infection) in age group $a$, during year $X$, is given by:

$$s_{a,X}=exp\left( -\sum_{i=0}^{a} \sum_{k=1}^{4} \lambda_{X-i}^{k} \right).$$

We assume that the force of infection (FOI) is time not serotype-specific, i.e. $\lambda_{X-i}^{k}=\lambda_{X-i}\left( k=1,2,3,4 \right)$, then the equation becomes

$$s_{a,X}=exp\left( -4\sum_{i=0}^{a} \lambda_{X-i} \right).$$

Now the fraction of people who experienced at least one infection is given by,

$$\pi_{a,X}=1-s_{a,X.}$$

The fraction of people who has experienced only one infection is given by,

$$\phi_{a,X}=4\left( 1-exp\left( -\sum_{i=0}^{a} \lambda_{X-i} \right) \right)exp\left( -3\sum_{i=0}^{a} \lambda_{X-i} \right).$$

Finally, the fraction of people who has experienced more than one infection is given by,

$$\alpha_{a,X}=1-s_{a,X}-\phi_{a,X}.$$

We use the sero-prevalence data obtained from sero-surveys done in 2013 in Singapore [13]. We have the number of participants and number of sero-positives for the age group 16–71.

The age group distribution in the sero-survey was: 16–20, 21–25, 26–30, 31–35, 36–40, 41–45, 46–50, 51–55, 56–60, and 60+ years.

A binomial log-likelihood is assumed for the FOI. The *optim* function in R used to find the maximum likelihood estimate of the FOI using the following equation:

$\mathcal{L}\left( \lambda_{X-i} \right)=\sum_{a} \left( N_{a,X}-P_{a,X} \right)\left( log\left( 1-\pi_{a,X} \right) \right)+P_{a,X}log\left( \pi_{a,X} \right)$,

where, $N_{a,X}$ is the total number of individuals in age group *a,* and $P_{a,X}$ is the number of seropositive individuals.

The average annual force-of-infection in respective periods 2008–2013, 2003–2007, ..., 1953–1957 are denoted by $\lambda^{p}$, $p=1,2,\ldots,12$. $\lambda^{13}$ represents the average annual FOI estimated for the years 1922–1952. The model fit with age-specific sero-prevalence data is presented in Fig C.


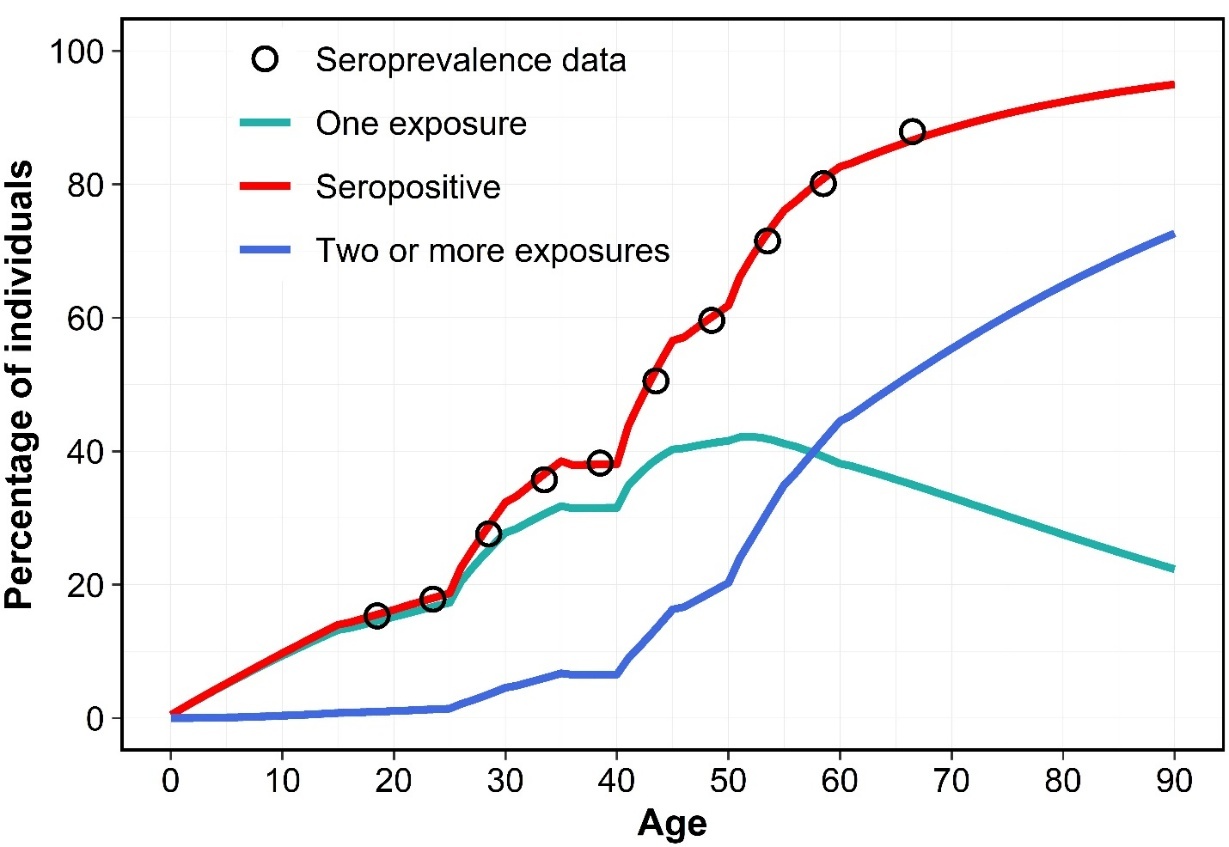


**Fig C:** The percentage of population with different exposure history with age. The solid lines are calculated from catalytic model and the black circles are age-specific sero-prevalence data, form the survey conducted in 2013 in Singapore.

# **Dengue surveillance data**


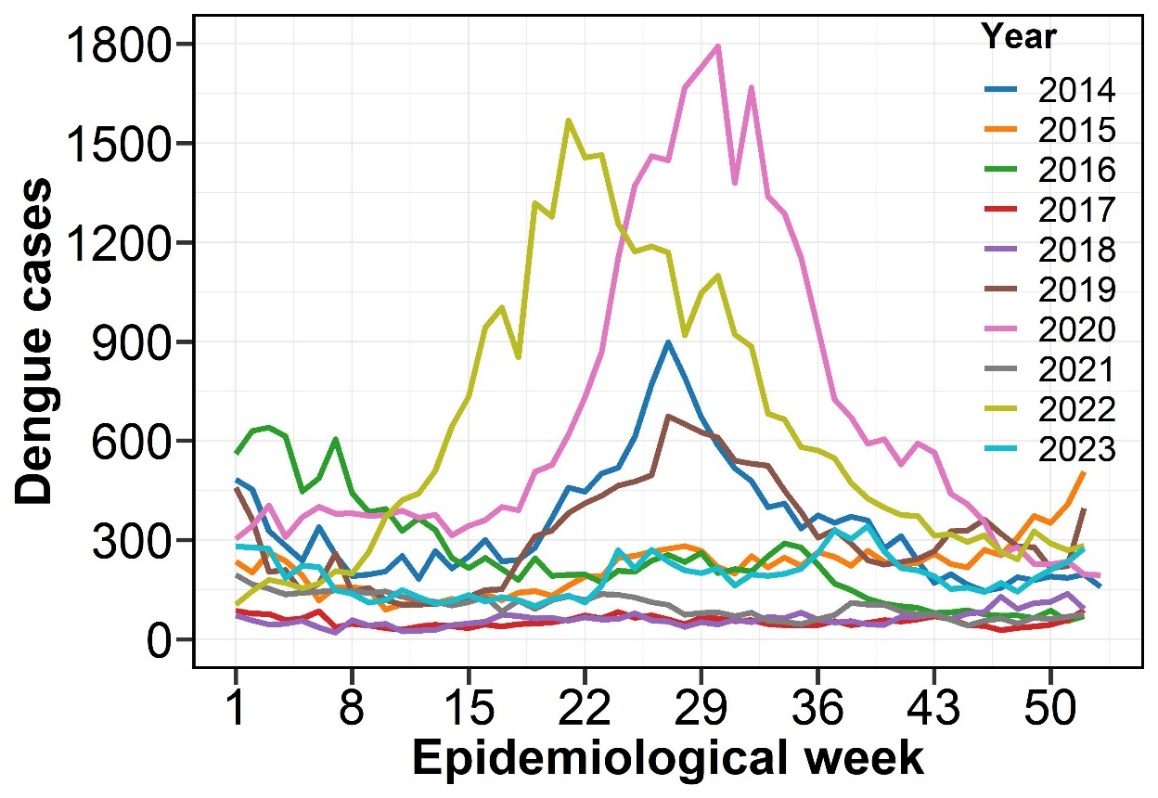


**Fig D**: Weekly dengue cases reported in Singapore during 2014-2023.


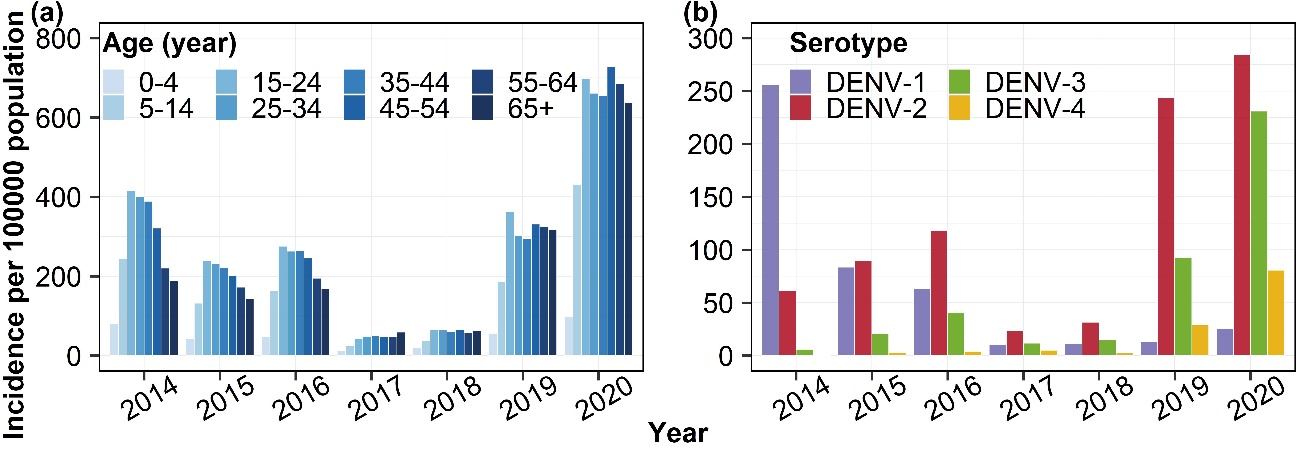


**Fig E:** The dengue incidence per 100000 population stratified according to (a) age groups and (b) serotypes, during 2014 to 2020 in Singapore.

# **Model fitting**


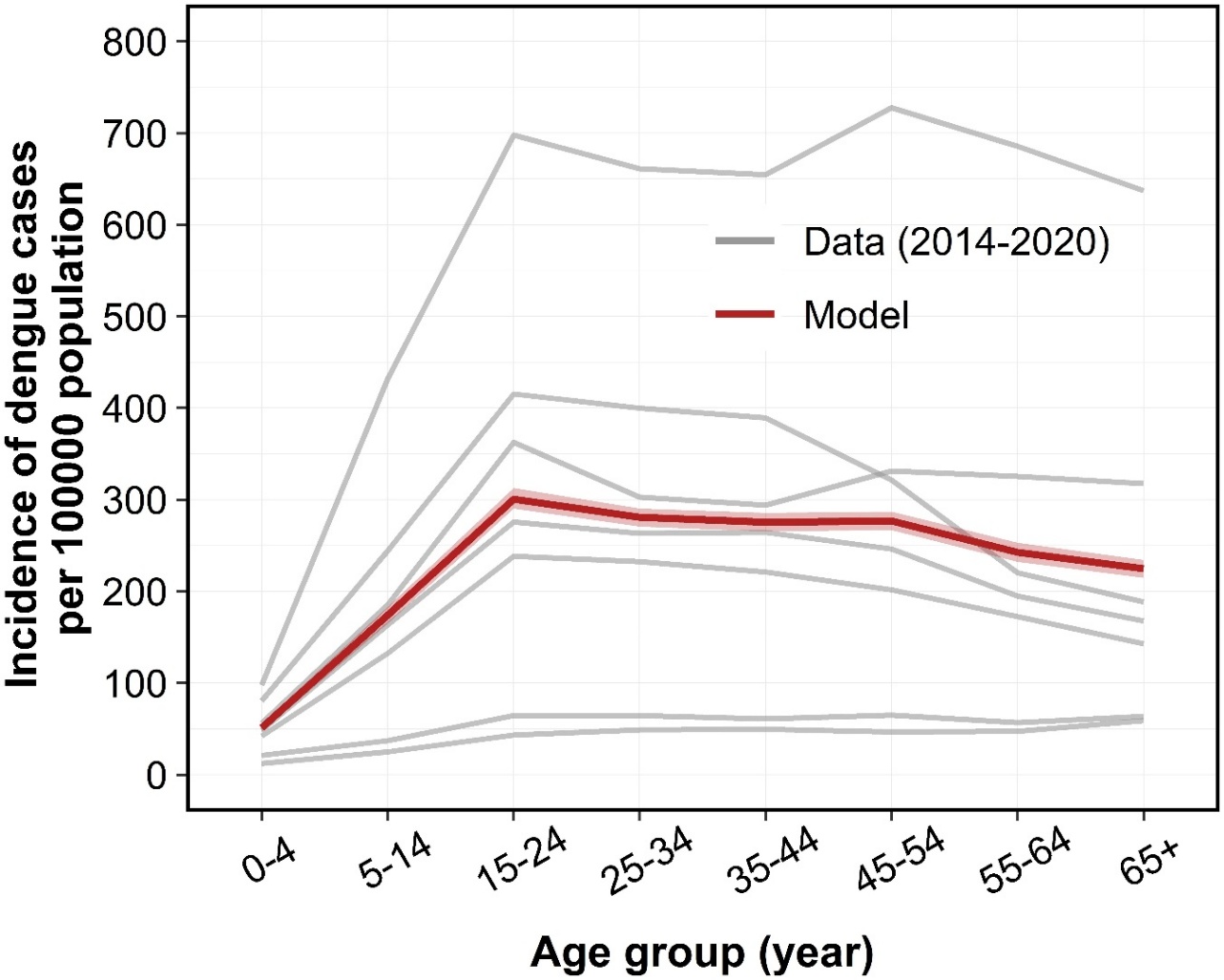


**Fig F**: Model fitting with data. The gray lines are the annual incidence of dengue cases per 100,000 population for each age group reported during 2014–2020. The dark red line is the median of incidence of cases per 100,000 population generated by the transmission model and the shaded region is the 95% credible interval.


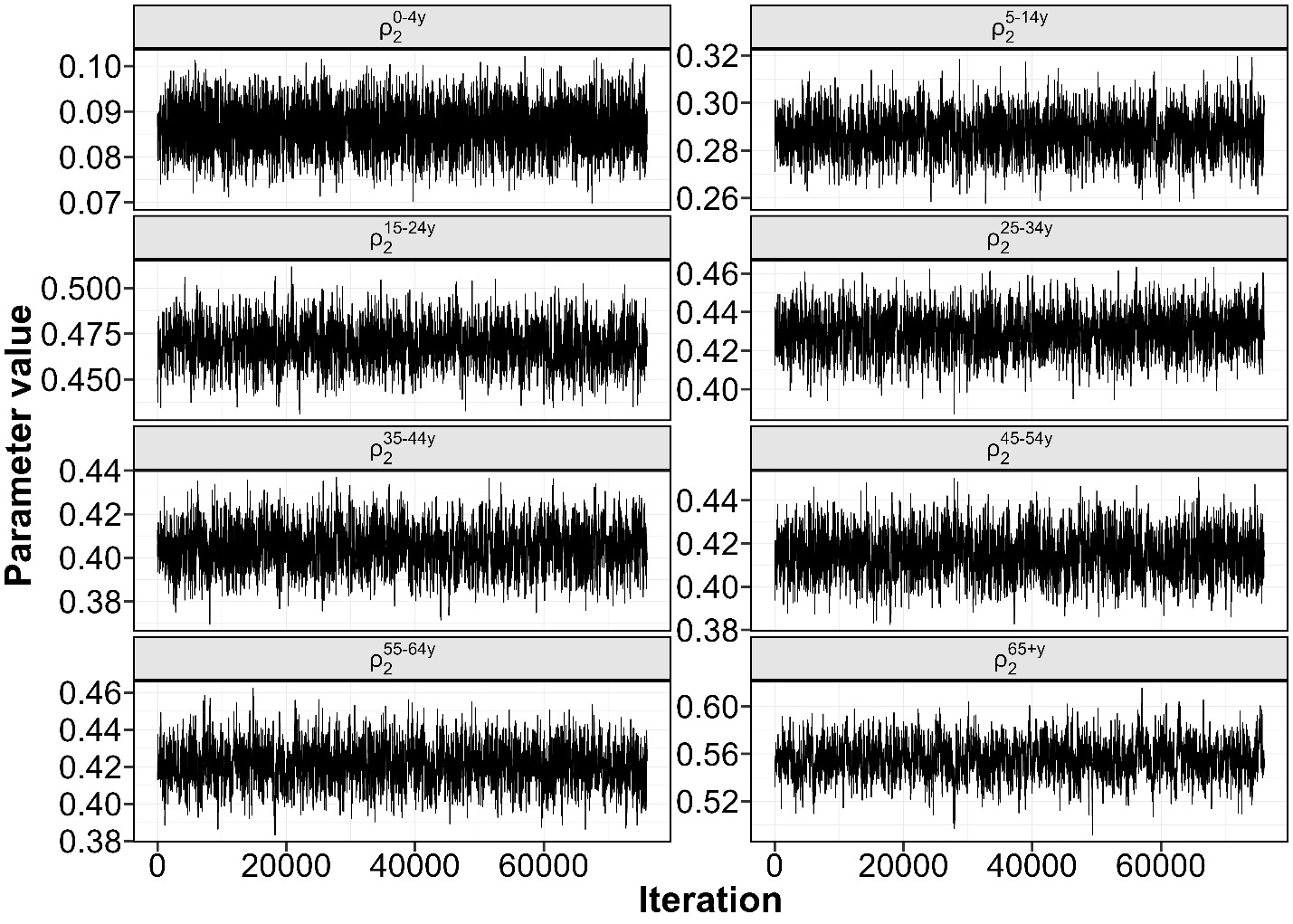
**Fig G:** Trace plot of the MCMC chain of the estimated parameters.

| **Parameter** | **Estimated value**  **Median (95% Credible Interval)** |
| --- | --- |
| $\rho_{2}^{0-4y}$ | 0·086 (0·077–0·095) |
| $\rho_{2}^{5-14y}$ | 0·286 (0·271–0·303) |
| $\rho_{2}^{15-24y}$ | 0·468 (0·448–0·489) |
| $\rho_{2}^{25-34y}$ | 0·429 (0·410–0·448) |
| $\rho_{2}^{35-44y}$ | 0·404 (0·386–0·423) |
| $\rho_{2}^{45-54y}$ | 0·415 (0·396–0·433) |
| $\rho_{2}^{55-64y}$ | 0·421 (0·401–0·441) |
| $\rho_{2}^{65+y}$ | 0·555 (0·527–0·583) |

**Table D:** Estimated value of age-specific fraction of secondary infection to be reported as dengue cases.

# **Hospitalization rate**

| **Parameter** | **Average hospitalization rate** |
| --- | --- |
| $\xi_{2}^{0-14y}$ | 0·38 |
| $\xi_{2}^{15-24y}$ | 0·36 |
| $\xi_{2}^{25-34y}$ | 0·33 |
| $\xi_{2}^{35-44y}$ | 0·39 |
| $\xi_{2}^{45-54y}$ | 0·46 |
| $\xi_{2}^{55-64y}$ | 0·52 |
| $\xi_{2}^{65+y}$ | 0·49 |

**Table E:** Average age-specific hospitalization rate. These data has been calculated from the data during 2007 to 2017, as reported in [5]**.**

# **Serotype distribution of dengue infection under dominating serotype scenarios**


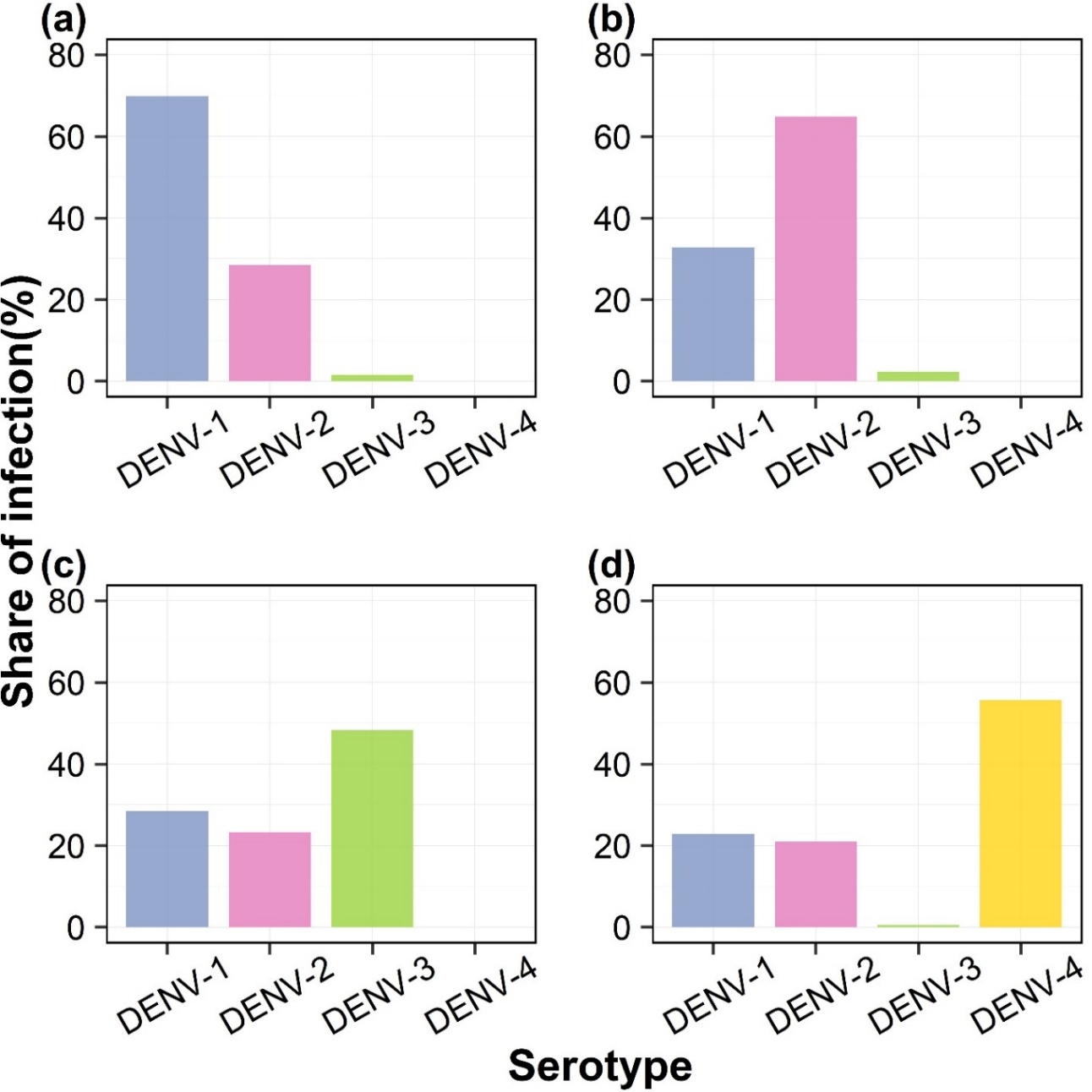


**Fig H:** Serotype distribution of dengue infection during the rollout of vaccine for four serotype-dominant scenarios: (a) DENV-1, (b)DENV-2, (c) DENV-3, and (d) DENV-4, for baseline scenario (without vaccination).

# **Sensitivity analysis**

**
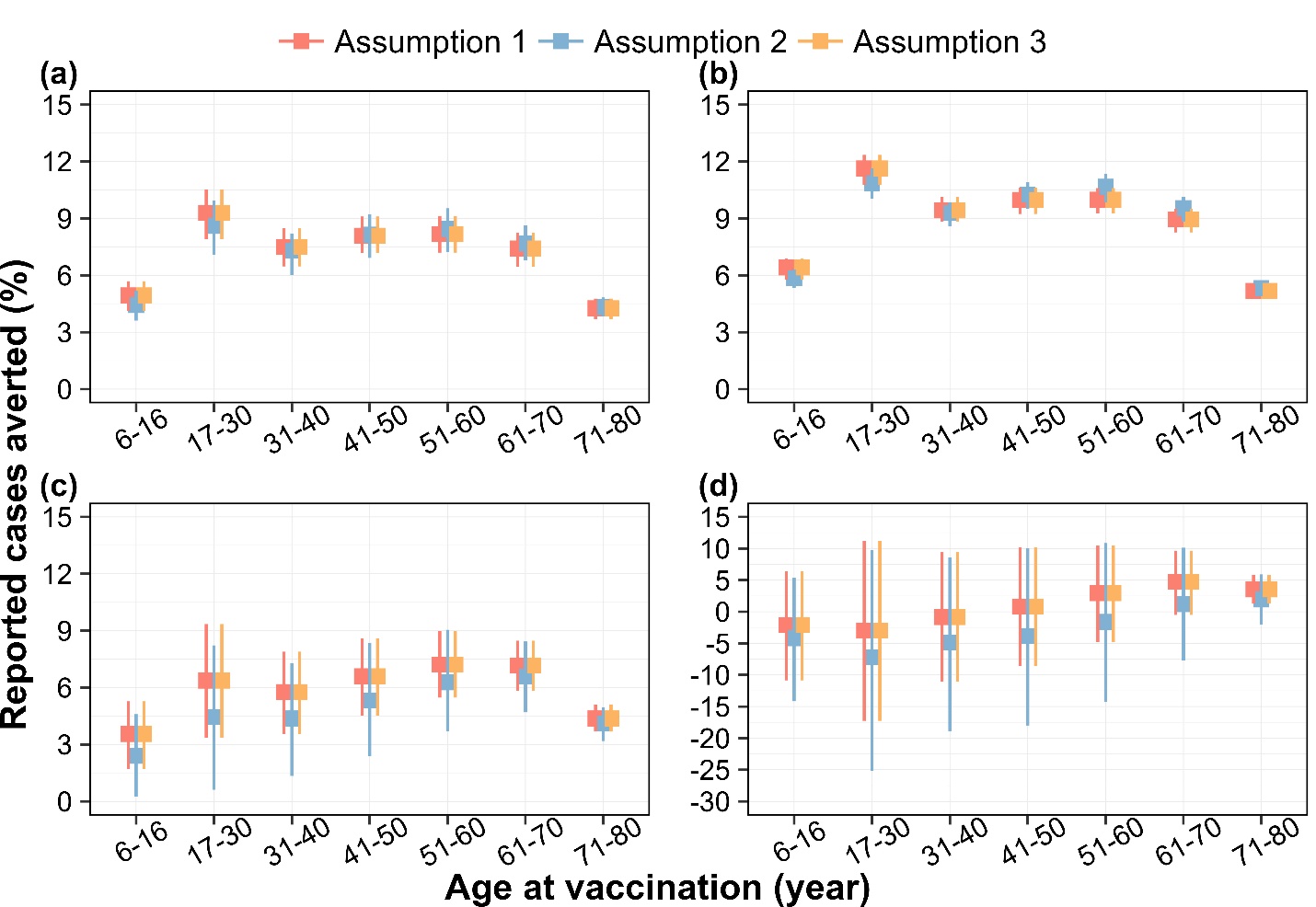
**

**Fig I:** Percentage of dengue cases averted by targeted age groups, over a 10-year routine vaccination program with Qdenga under (a) DENV-1, (b) DENV-2, (c) DENV-3, and (d) DENV-4 dominant scenarios, for three different assumptions. Assumption 1 is the baseline assumption (presented in the main text) that both age-specific reporting rate and hospitalization rates depend on the pre-exposure history of infection, reporting rate among secondary infection is twice of that of primary infection; the hospitalization rate among secondary infection is 4 times higher than that of primary infection. In assumption 2, both reporting rate and hospitalization rate are only age-stratified but does not depend on pre-exposure history. In assumption 3, the reporting rate follows the same assumption as in assumption 1 but the hospitalization rate does not depend on pre-exposure history. All the estimates presented are for vaccine coverage 80%. Solid squares represent the mean model projections, and error bars indicate the corresponding 95% range of simulations.

**
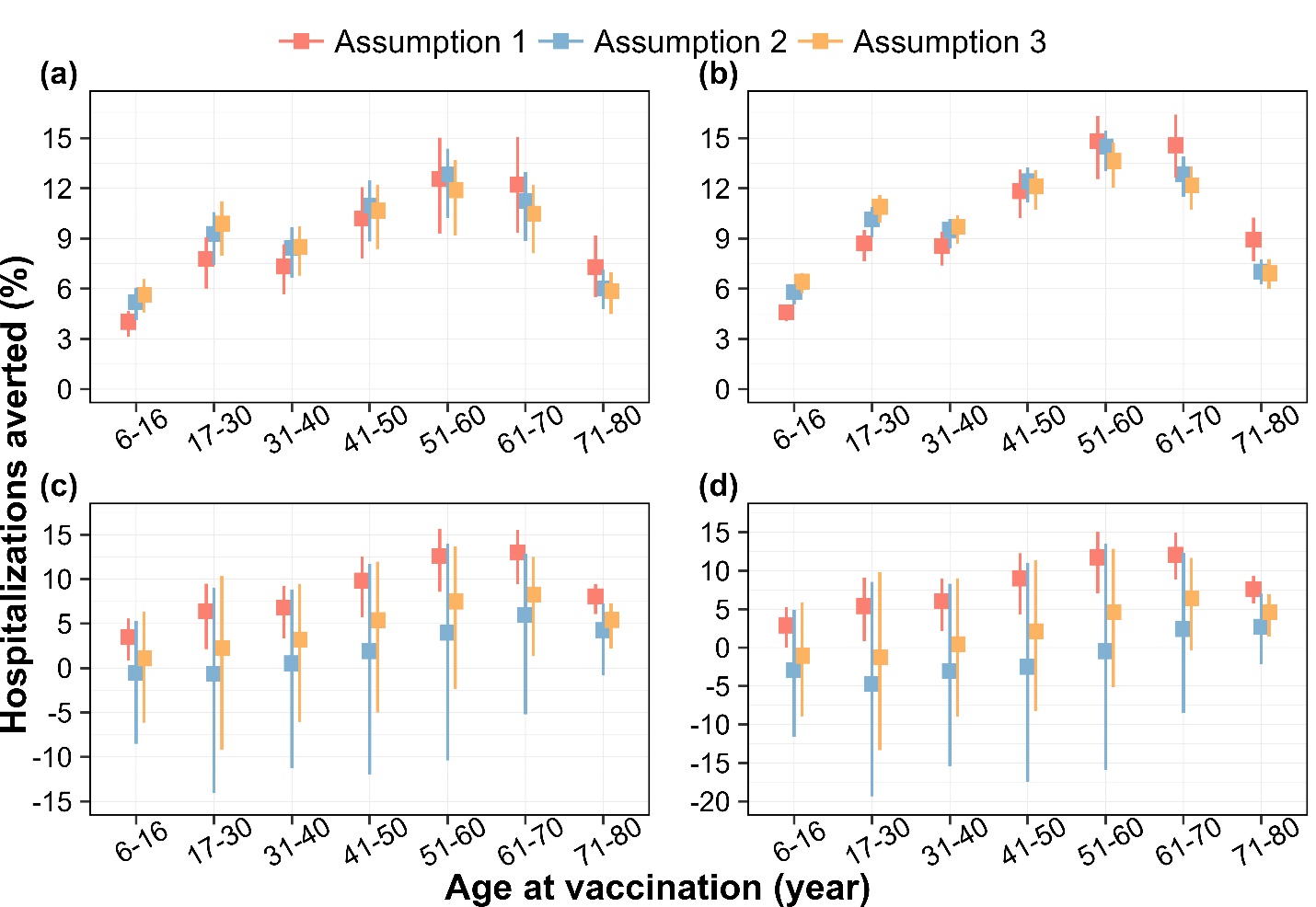
**

**Fig J:** Percentage of hospitalizations averted by targeted age groups, over a 10-year routine vaccination program with Qdenga under (a) DENV-1, (b) DENV-2, (c) DENV-3, and (d) DENV-4 dominant scenarios, for three different assumptions. Assumption 1 is the baseline assumption (presented in the main text) that both age-specific reporting rate and hospitalization rates depend on the pre-exposure history of infection, reporting rate among secondary infection is twice of that of primary infection; the hospitalization rate among secondary infection is 4 times higher than that of primary infection. In assumption 2, both reporting rate and hospitalization rate are only age-stratified but does not depend on pre-exposure history. In assumption 3, the reporting rate follows the same assumption as in assumption 1 but the hospitalization rate does not depend on pre-exposure history. All the estimates presented are for vaccine coverage 80%. Solid squares represent the mean model projections, and error bars indicate the corresponding 95% range of simulations.

# **Number of dengue cases and hospitalizations averted per 1000 vaccination**


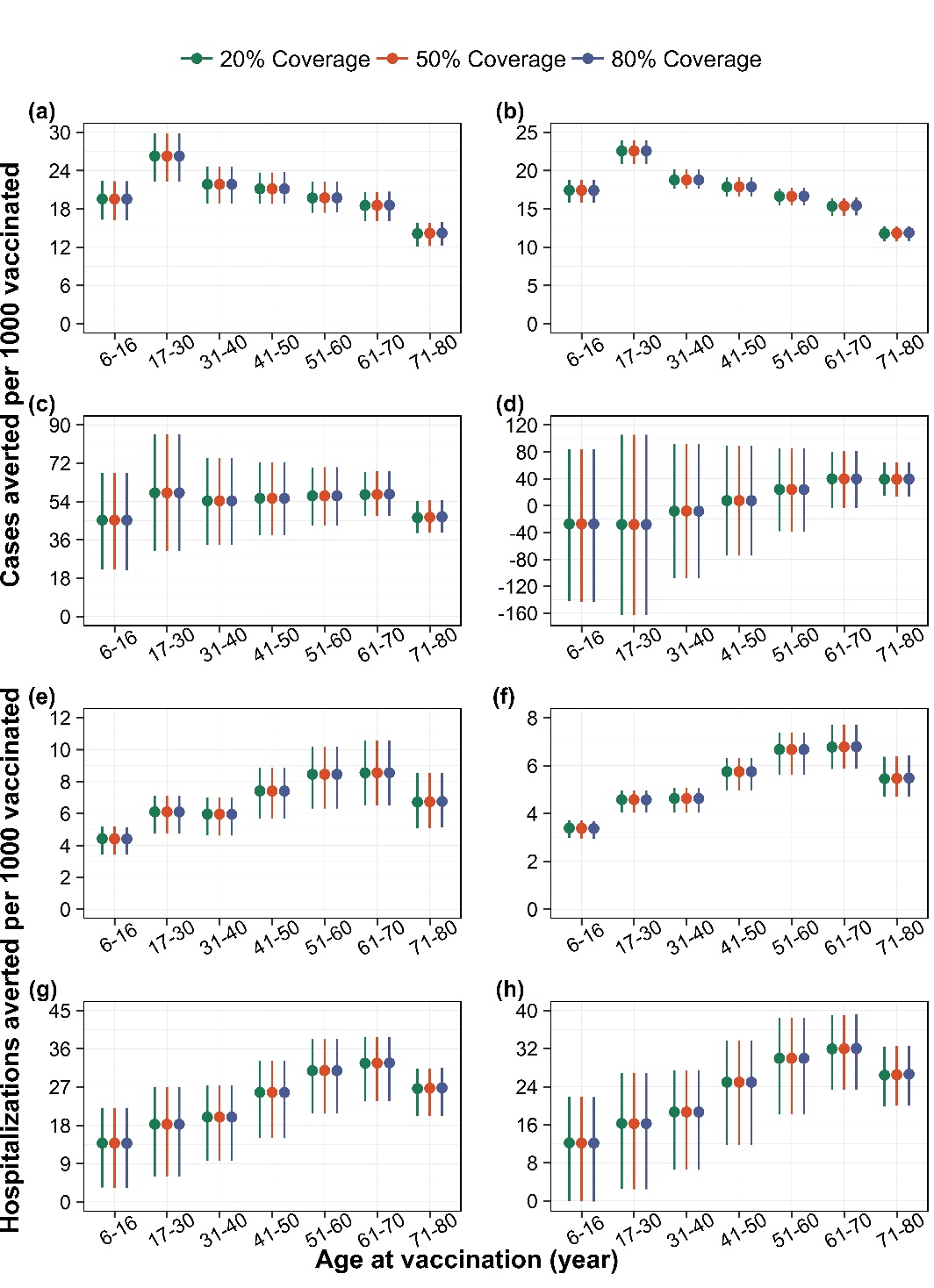


**Fig K:** Absolute number of reported dengue cases averted per 1000 vaccinated by targeted age groups under (a) DENV-1, (b) DENV-2, (c) DENV-3, and (d) DENV-4 dominant scenarios, and absolute number of hospitalizations due to dengue averted per 1000 vaccinated by targeted age groups under (e) DENV-1, (f) DENV-2, (g) DENV-3, and (h) DENV-4 dominant scenarios, for three different vaccine coverage levels, over a 10-year routine vaccination program with Qdenga. Solid dots represent the mean model projections, and error bars indicate the corresponding 95% range of simulations.

# **Impact of vaccination in different age groups**


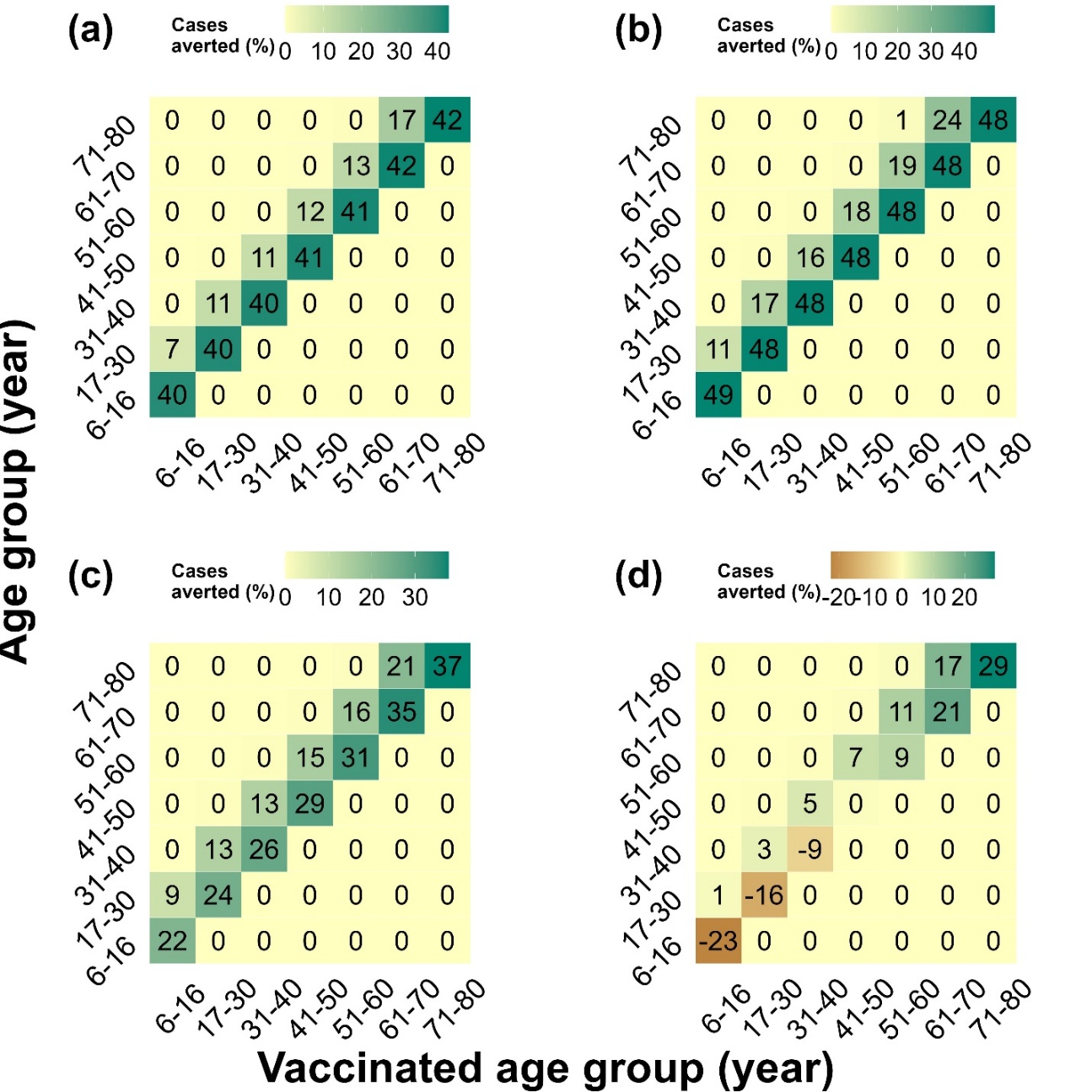


**Fig L:** Mean estimate of impact of vaccination on different age-groups in terms of percentage averted reported cases in each age groups, under (a) DENV-1, (b) DENV-2, (c) DENV-3, and (d) DENV-4 dominant scenarios. The horizontal axis denotes different targeted age groups, and the vertical axis denotes the age groups for which the impact has been estimated. All the estimates presented are for vaccine coverage 80%.


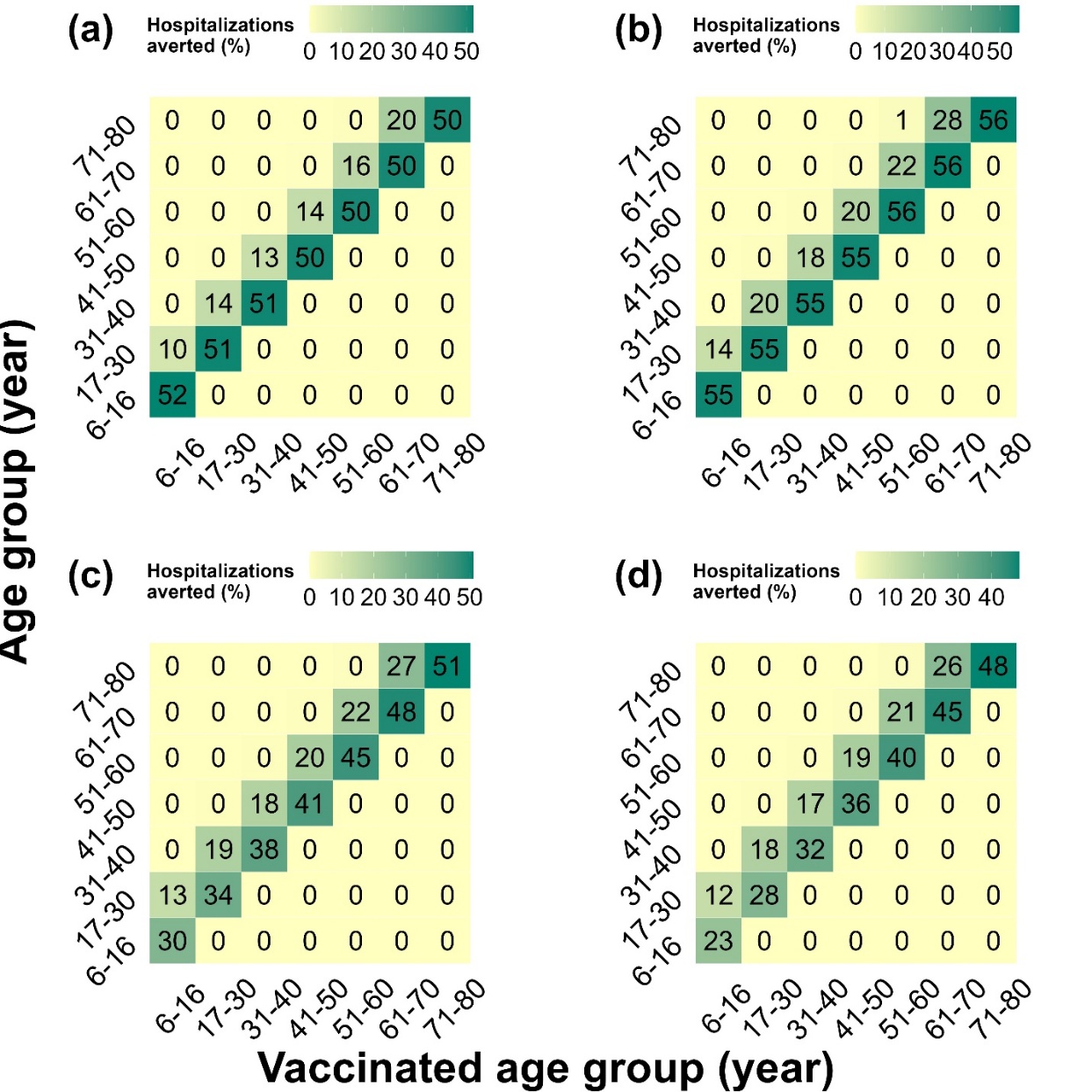


**Fig M:** Mean estimate of impact of vaccination on different age-groups in terms of percentage of averted hospitalizations in each age groups, under (a) DENV-1, (b) DENV-2, (c) DENV-3, and (d) DENV-4 dominant scenarios. The horizontal axis denotes different targeted age groups, and the vertical axis denotes the age groups for which the impact has been estimated. All the estimates presented are for vaccine coverage 80%.

# **Impact of vaccination with alternative targeted age-groups**


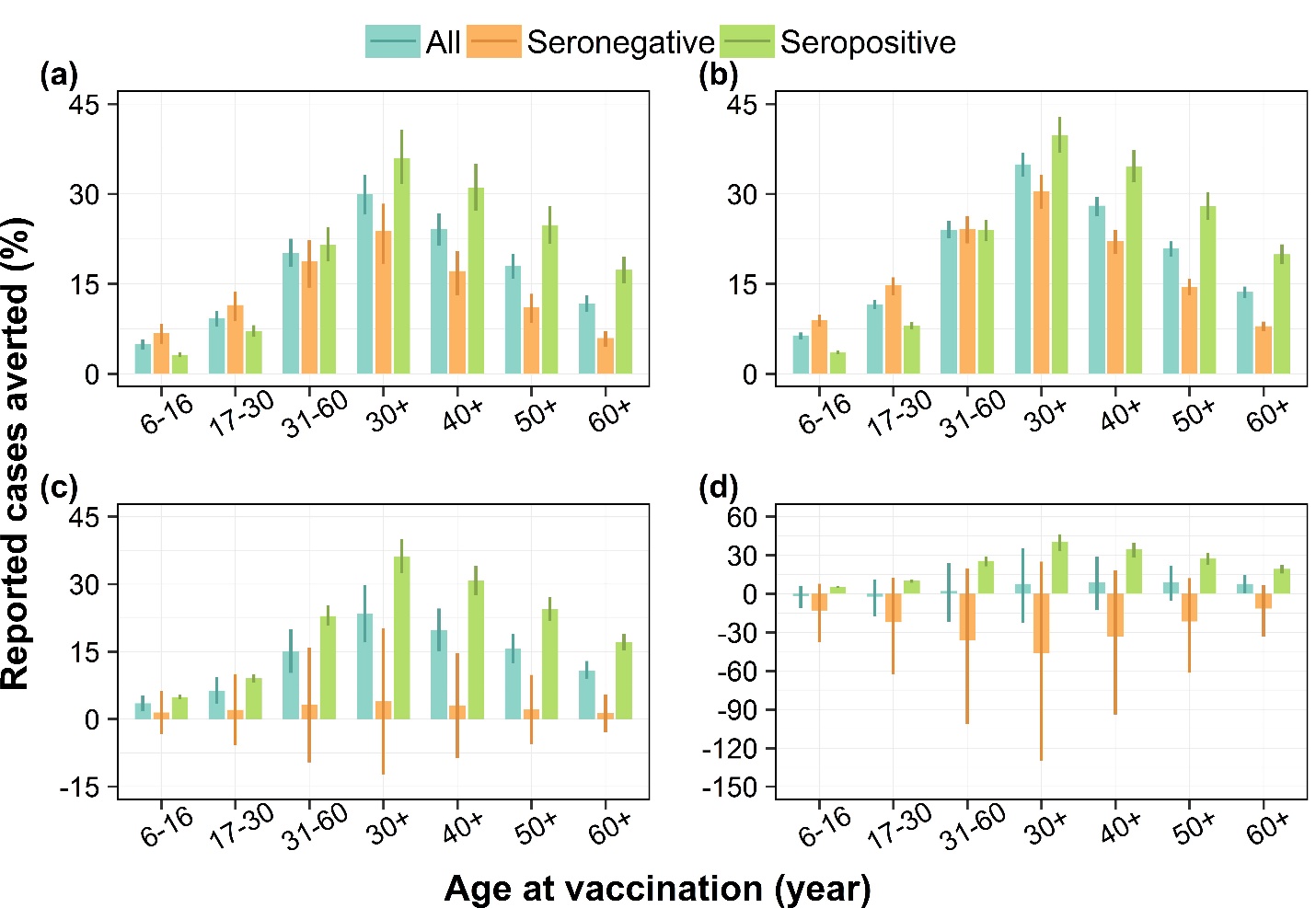


**Fig N:** Percentage of reported dengue cases averted by targeted age groups over a 10-year routine vaccination program under (a) DENV-1, (b) DENV-2, (c) DENV-3, and (d) DENV-4 dominant scenarios, for the whole population, seropositive individuals only, and seronegative individuals only, assuming 80% vaccine coverage. Bars represent mean model estimates, and error bars indicate the corresponding 95% range of simulations.


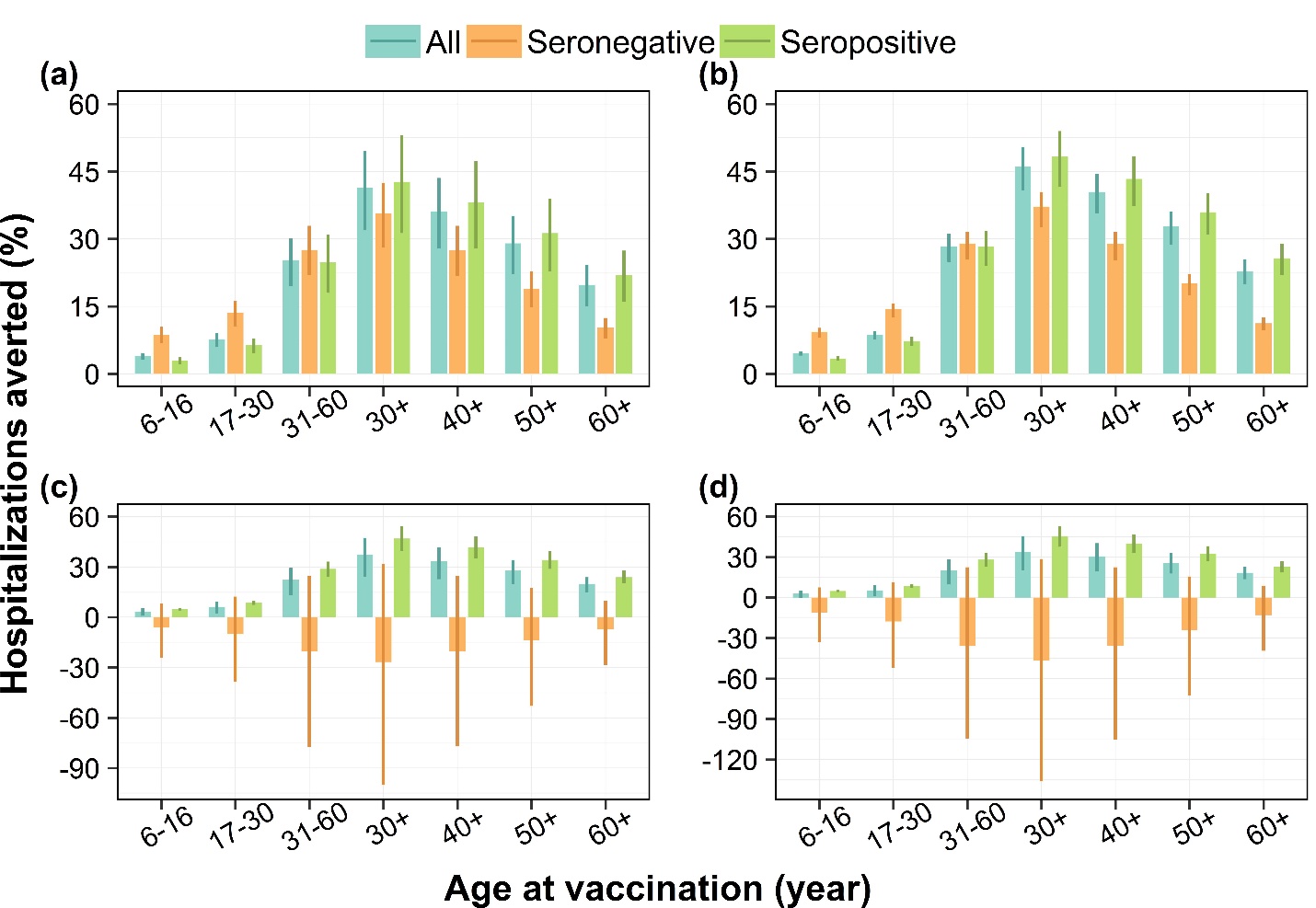


**Fig O:** Percentage of reported hospitalizations due to dengue averted by targeted age groups over a 10-year routine vaccination program under (a) DENV-1, (b) DENV-2, (c) DENV-3, and (d) DENV-4 dominant scenarios, for the whole population, seropositive individuals only, and seronegative individuals only, assuming 80% vaccine coverage. Bars represent mean model estimates, and error bars indicate the corresponding 95% range of simulations.


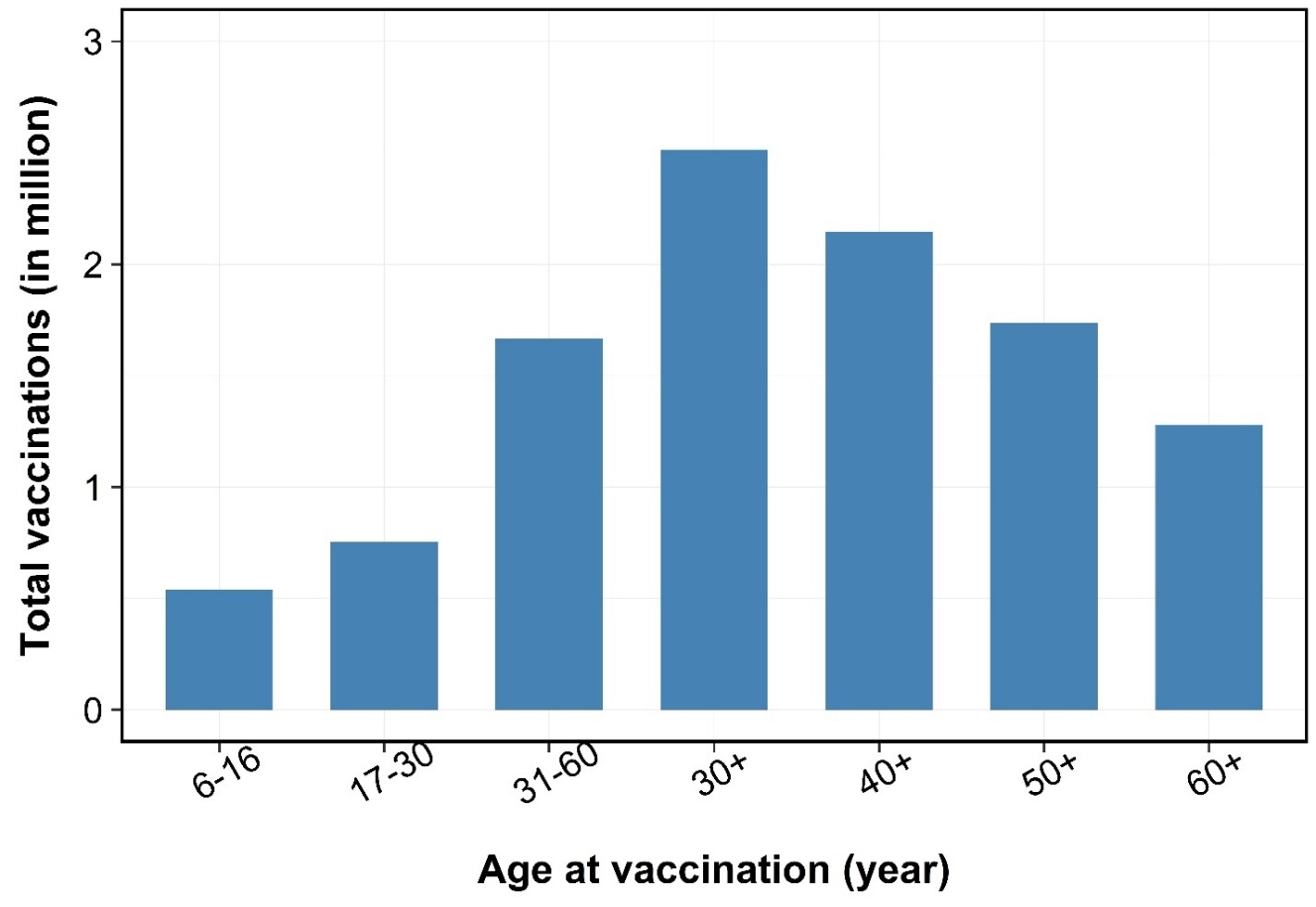


**Fig P:** Total number of vaccinations for different targeted age groups. In each of the targeted age groups the coverage is 80%.

# **References**

1. Singapore Department of Statistics. Singapore Population. 2023 [cited 4 Mar 2024]. Available: http://www.singstat.gov.sg/modules/infographics/population

2. United Nations. World Population Prospects. 2023 [cited 4 Mar 2024]. Available: https://population.un.org/wpp/Graphs/DemographicProfiles/Line/702

3. Ferguson NM, Rodríguez-Barraquer I, Dorigatti I, Mier-y-Teran-Romero L, Laydon DJ, Cummings DAT. Benefits and risks of the Sanofi-Pasteur dengue vaccine: Modeling optimal deployment. Science. 2016;353: 1033–1036. doi:10.1126/science.aaf9590

4. Flasche S, Jit M, Rodríguez-Barraquer I, Coudeville L, Recker M, Koelle K, et al. The Long-Term Safety, Public Health Impact, and Cost-Effectiveness of Routine Vaccination with a Recombinant, Live-Attenuated Dengue Vaccine (Dengvaxia): A Model Comparison Study. Von Seidlein L, editor. PLoS Med. 2016;13: e1002181. doi:10.1371/journal.pmed.1002181

5. Ang LW, Thein T-L, Ng Y, Boudville IC, Chia PY, Lee VJM, et al. A 15-year review of dengue hospitalizations in Singapore: Reducing admissions without adverse consequences, 2003 to 2017. Althouse B, editor. PLoS Negl Trop Dis. 2019;13: e0007389. doi:10.1371/journal.pntd.0007389

6. Murgue B, Roche C, Chungue E, Deparis X. Prospective study of the duration and magnitude of viraemia in children hospitalised during the 1996-1997 dengue-2 outbreak in French Polynesia. J Med Virol. 2000;60: 432–438. doi:10.1002/(SICI)1096-9071(200004)60:4<432::AID-JMV11>3.0.CO;2-7

7. Anderson KB, Gibbons RV, Cummings DAT, Nisalak A, Green S, Libraty DH, et al. A Shorter Time Interval Between First and Second Dengue Infections Is Associated With Protection From Clinical Illness in a School-based Cohort in Thailand. The Journal of Infectious Diseases. 2014;209: 360–368. doi:10.1093/infdis/jit436

8. Whitmire RE, Burke DS, Nisalak A, Harrison BA, Watts DM. Effect of Temperature on the Vector Efficiency of Aedes aegypti for Dengue 2 Virus. The American Journal of Tropical Medicine and Hygiene. 1987;36: 143–152. doi:10.4269/ajtmh.1987.36.143

9. Salazar MI, Richardson JH, Sánchez-Vargas I, Olson KE, Beaty BJ. Dengue virus type 2: replication and tropisms in orally infected Aedes aegypti mosquitoes. BMC Microbiol. 2007;7: 9. doi:10.1186/1471-2180-7-9

10. Sheppard PM, Macdonald WW, Tonn RJ, Grab B. The Dynamics of an Adult Population of Aedes aegypti in Relation to Dengue Haemorrhagic Fever in Bangkok. The Journal of Animal Ecology. 1969;38: 661. doi:10.2307/3042

11. Olivera-Botello G, Coudeville L, Fanouillere K, Guy B, Chambonneau L, Noriega F, et al. Tetravalent Dengue Vaccine Reduces Symptomatic and Asymptomatic Dengue Virus Infections in Healthy Children and Adolescents Aged 2–16 Years in Asia and Latin America. J Infect Dis. 2016;214: 994–1000. doi:10.1093/infdis/jiw297

12. Tricou V, Yu D, Reynales H, Biswal S, Saez-Llorens X, Sirivichayakul C, et al. Long-term efficacy and safety of a tetravalent dengue vaccine (TAK-003): 4·5-year results from a phase 3, randomised, double-blind, placebo-controlled trial. The Lancet Global Health. 2024;12: e257–e270. doi:10.1016/S2214-109X(23)00522-3

13. Tan LK, Low SL, Sun H, Shi Y, Liu L, Lam S, et al. Force of Infection and True Infection Rate of Dengue in Singapore: Implications for Dengue Control and Management. American Journal of Epidemiology. 2019;188: 1529–1538. doi:10.1093/aje/kwz110
